# Polydopamine surface functionalized submicron ZnO for broadening the processing window of 3D printable PLA composites

**DOI:** 10.1007/s10965-023-03540-w

**Published:** 2023-04-14

**Authors:** Xiao-Mei Yang, Guang-Zhong Yin, Olga Zafra Amorós, María Arroyo Hernández, Jimena de la Vega, José Manuel Torralba

**Affiliations:** 1 IMDEA Materials Institute, C/Eric Kandel, 2, Getafe, 28906 Spain; 2 Escuela Politécnica Superior, Francisco de Vitoria University (UFV), Ctra. Pozuelo-Majadahonda Km 1,800, Pozuelo de Alarcón, 28223 Spain; 3 Faculty of Experimental Sciences, Francisco de Vitoria University (UFV), Ctra. Pozuelo-Majadahonda Km 1,800, Pozuelo de Alarcón, 28223 Spain; 4 Universidad Carlos III Madrid, Av. Universidad 30, Leganés, 28011 Spain

**Keywords:** PLA, Surface functionalization, ZnO, 3D printing, PDA

## Abstract

The “catalytic degradation” of metal oxides limits the wide application of PLA when PLA needs to be modified by adding metal oxides to achieve desired properties. Zinc oxide (ZnO) is a common and widely used agent as it can be used for many properties, such as antioxidant, antibacterial, etc. However, detrimental effects often exist on the properties of polymers after introducing the ZnO, due to the catalytic degradation. In this study, we used polydopamine (PDA) to construct ZnO@PDA core-shell submicron particles via the self-polymerization of dopamine (DA) in alkaline solution, aimed to produce a surface functionalization that would be used to control the rate of degradation of PLA by ZnO during thermal processing, and promote the preservation of mechanical properties. PLA with different contents of ZnO and ZnO@PDA were prepared by a simple melt extrusion method. The degradation behavior, mechanical properties and antibacterial activity of ZnO/PLA and ZnO@PDA/PLA were investigated. It was found that the incorporation of ZnO@PDA in PLA at different contents exhibits a dramatic control over the degradation rate when compared to that of the ZnO/PLA with the same filler content. Notably, the T_5%_ and T_50%_ of 3%-ZnO@PDA/PLA increased by 36.4 ^o^C and 31.9 ^o^C. GPC results showed the molecular weight of 3%-ZnO@PDA/PLA was only reduced by 15.8% after thermal processing. In addition, 3%-ZnO@PDA/PLA can be 3D-printed smoothly. That is to say, the introduction of ZnO@PDA can increase the processing window of PLA/ZnO composites, providing the possibility for materials that need to be included in civil application. Accordingly, ZnO@PDA/PLA samples showed higher tensile strength and elongation at break than that of corresponding ZnO/PLA samples. Regarding the antibacterial behavior, the ZnO@PDA/PLA have more bacterial growth disability effect against Gram(+) bacteria than that of pure PLA.

## Introduction

Polylactic acid (PLA), as an environmentally friendly polymer, become one of the most promising candidates in civil products due to its biocompatible with superior mechanical properties, good processability and low cost [1]. In addition, PLA has distinct advantages like low energy consumption and emission of low greenhouse gas during production and is suitable for 3D printing applications [2]. As a linear aliphatic thermoplastic polyester, PLA produced by biomass lactic acid has been widely concerned mainly because of its sustainability and easily biodegradable character [3].

Metal oxides play a very important role in world science applications. For example, ZnO can be used for the fabrication of microelectronic circuits, solar energy conversion, sensors, electrochromic device applications, piezoelectric devices, semiconductors, and catalysis [4]. However, a significant problem is that when ZnO particles are introduced as modifiers to improve the properties of PLA such as antioxidant and antibacterial ones, whether micron, submicron, or nanoscale, ZnO particles will severely degrade PLA. The addition of ZnO has a deleterious effect on PLA. Usually, it can lead to an intense degradation of the PLA chains during melting processing, with a consequent substantial reduction of molecular weights so as to thermomechanical properties. The scientists adopted some technical methods to overcome this issue. For example, Murario et al. have demonstrated that it is possible to obtain competitive composites when the PLA/ZnO interface is adequately tuned, via silane surface treatment [5]. However, as a metal-inorganic filler, ZnO has poor compatibility with organic polymer materials. Hence, the polymerization of functional polymers such as hydrophilic poly-(ethylene terephthalate) (PET) [6], polypyrrole [7], polyaniline [8], etc. on ZnO surface could be a good way to improve the interface, but the process normally requires harsh conditions, for example it needs a low pH (2 ~ 3) with an acidic modifying agent, which will harmful to ZnO (add reference). Therefore, a possible solution is to use an additional redox agent for triggering polymerization at neutral pH or to deposit polymers, synthesized at mild condition, e.g., pH (8 ~ 10).

Among the polymers of interest, polydopamine (PDA) is a very attractive compound. PDA is a bio-resource high adhesive material that has been used as a versatile coating and platform for biomedical application, drug delivery system preparation, sensors and biosensors, energy storage and batteries as well as photocatalysis [9–14]. One of the most important advantages of PDA is its strong adhesive properties that allows to coat any surface both hydrophilic and hydrophobic. So far, PDA has been used to coat many materials i.e. alumina [15], silica [16], magnetite [17], wood [18], glass [19], nanodiamonds [20], and other carbon based materials [21]. The crucial attribute allowing to use of PDA in different applications is its biocompatibility that has been proved under in vitro and in vivo conditions [22, 23]. Routinely, PDA is obtained by oxidative polymerization of dopamine in Tris-buffer at pH 8.5, which could be very promising for ZnO coating. Therefore, in this work, PDA was used to modify submicron ZnO, and then ZnO before and after modification were added to PLA by melt extrusion. The degradation behavior during heat processing and bacteriostatic properties of ZnO before and after modification on PLA will be studied and discussed.

## Experimental section

### Materials

Poly (lactic acid) grade Ingeo™ 2003D manufactured by Nature Works^®^ (Minnetonka, MN) was used as a polymer matrix. Dopamine hydrochloride (98%) was purchased from Merck. 1,1,1-Tris(4-hydro-xyphenyl) ethane (> 98.0% (GC)) was purchased from TCI. Hydrochloric acid (37%) was purchased from Sigma-Aldrich. ZnO (< 5 μm particle size, 99.9%) was purchased from Aladdin.

### Fabrication of ZnO@PDA particles

The ZnO@PDA particles were synthesized according to the following steps. The first step involves the preparation of the Tris solution and ZnO dispersion: deionized water (500 mL) was mixed with Tris (6.07 g) and ZnO (26 g) in clean beakers respectively by vigorous magnetic stirring. After stirring for 30 min, ZnO dispersion was poured into the Tris solution, and adjusting the pH to 8.5 with concentrated hydrochloric acid. The second step is adding 1.5 g dopamine hydrochloride into the above solution with magnetic stirring, and the stirring was kept at room temperature for another 24 h. The gray dispersions were centrifuged and washed with deionized water at least 5 times, and with ethanol at last. Then the powders were transferred into vacuum oven at 60 ^o^C for 24 h. Finally, the ZnO@PDA particles were obtained. The PDA particles were synthesized using a similar procedure without participation of ZnO particles.

### Preparation of PLA composites

PLA composites were prepared from a melt extrusion processing method. In brief, the ZnO/PLA and ZnO@PDA/PLA samples premixed samples were prepared in a Twin-screw extruder (Brabender) at 180 ^o^C, at a mixing speed of 52 rpm. In order to be compared at the same conditions, the pure PLA was also prepared in a melt extrusion mixer. The obtained filaments were chopped into small granules to the approximate size of PLA pellets using a pelletizer (Granulator, Brabender). Prior to melt mixing, PLA was dried in an oven at 80 ^o^C for 24 h to remove the excess of moisture. In this paper, the samples were tagged as “x-ZnO/PLA, and x-ZnO@PDA/PLA”, where x represented the adding ratio of ZnO or ZnO@PDA to PLA matrix, respectively.

### Physical-chemical characterization

Fourier transform infrared (FTIR) spectra of ZnO, PDA, ZnO@PDA particles, and pure PLA, ZnO/PLA and ZnO@PDA/PLA composites were measured by using a FTIR spectrometer (Nicolet Is50, Thermo Fisher Scientific, S.L.U., USA) in the attenuated total reflection (ATR) mode. Transmission electron microscope (TEM) images of the ZnO and ZnO@PDA were observed via a microscope instrument (FEI Europe B.V., Netherlands). Particle size measurements of ZnO and ZnO@PDA were performed using a particle size analyzer (Bettersizer ST, China). The weight-average molecular weight (M_w_) and number-average molecular weight (M_n_) of PLA and PLA composites after extrusion were measured by gel permeation chromatography (GPC) with four columns (Waters 1525 Binary HPLC Pump) equipped with a Refractive Index Detector (IR, Waters 2414) and a series of narrow polystyrene standards with THF as mobile phase at 30 ^o^C.

The X-ray diffraction (XRD) patterns of the ZnO and ZnO@PDA particles were collected and analyzed using X-ray diffractometer (Panalytical B.V, Almelo, EA, Netherlands) with Cu Kα (λ = 1.54 Å) radiation as the source in the 2θ range of 5 ~ 80 ^o^. The thermal stability of the pure ZnO, ZnO@PDA, and PLA and PLA composites was evaluated using a thermogravimetric analyzer (Q50, TA Instruments). The measurements were performed in the range of 40 to 800 ^o^C at a heating rate of 10 ^o^C/min in nitrogen atmosphere. The mass of the samples was about 10 mg. The crystallization and melting properties of the PLA/ZnO and ZnO@PDA/PLA composites, such as glass transition temperatures (T_g_), crystallization temperatures (T_c_), melting temperatures (T_m_) were evaluated by differential scanning calorimetric (DSC) (Q200, Q200, TA Instruments). The degree of crystallinity ($\phi$ %) for samples was calculated according to the following Eq. (1):

1 $$\phi\, \%=\frac{\varDelta {H}_{m}-\varDelta {H}_{c}}{\omega {\varDelta H}_{m}^{0}}\times 100\, \%$$

where $\varDelta {H}_{m}$ and $\varDelta {H}_{c}$ are the enthalpies of melting and cold crystallization, respectively. ω and ${\varDelta H}_{m}^{0}$ are the weight fraction of PLA and melting enthalpy of 100% crystalline PLA (93.7 J/g), respectively [24]. Scanning electron microscopy (SEM) images of the fractured surfaces of the pure and PLA composites were obtained using scanning electron microscope (FEI Europe B.V., Netherlands) to examine the fracture morphology of the PLA and PLA composites. Before imaging, all samples were sputtered with a conductive gold thin layer with sputter current 30 mA and sputter time 60 s before observation. Stress-strain curves of pure PLA and PLA composites were measured in tensile tests (according to ASTM method D882-88 standard procedure) by an Instron 5966 machine equipped with a 2000 N capacity load cell. Samples with dimensions of 50 mm × 5 mm × 2 mm were cut from the films made by hot-press at 170 ^o^C and clamped by two pneumatic grips to study their mechanical behavior. The initial spacing between the upper and lower grips was fixed to 50 mm, and the strain rate was set to 2 mm/min. The splines were stretched until complete failure. Tensile tests were repeated five times per each set of splines. The 3D filament was made by 3 Devo filament maker, the screw temperature is 180 ^o^C and the diameter of the filament is about 2.85 mm. 3D printing sample was prepared by layer-by-layer fused deposition modeling process with the printing (Ultimaker S5) temperature 200 ^o^C and the build plate temperature 60 ^o^C.

### Antimicrobial activity characterization

The antimicrobial activity of the sample against bacterial pathogens was determined by direct contact with the composites according to the ISO 22,196: 2011. Two test microorganisms, *Staphylococcus aureus* (*S. aureus*) 12600 strain (from American Type Culture Collection) suitable for quality controls and *Escherichia coli* (*E. coli*) 35218 strain (from American Type Culture Collection) suitable for sensitivity assays, were used as models for Gram-positive bacteria and Gram-negative bacteria, respectively. Before antimicrobial test, each bacterium was grown in LB broth (Lennox) (NZYTech) at 37 ^o^C for 24 h. After that, culture was diluted, with the same broth, at approximately 10^6^ − 10^7^ CFU/mL and 0.4 mL were placed in the test surface and covered with 40 mm × 40 mm× 0.1 mm aseptic film. Each test was placed in a sterilized petri dish and cultivated at 37 ^o^C for 24 h in a moisturized environment. Following ISO 22196:2011 procedure, bacteria were recovered adding 10 mL of LB broth and viable count were done by serial dilutions on LB broth and plating on LB plus 1.5% agar (CondaLab). After 24 h of growing at 37 ^o^C, colonies were counted, and CFU/ml were calculated. Each material/bacterium were tested four times and two technical replicas were done each time. Statistical calculations were done using the R-software.

## Results and discussion

### Surface modification of ZnO

**Fig. 1:**
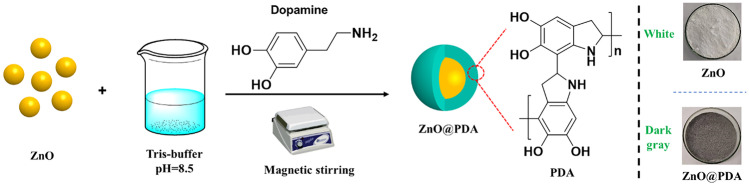
Synthesis route of ZnO@PDA, and the images of ZnO (white powder) and ZnO@PDA (dark gray powder)

**Fig. 2:**
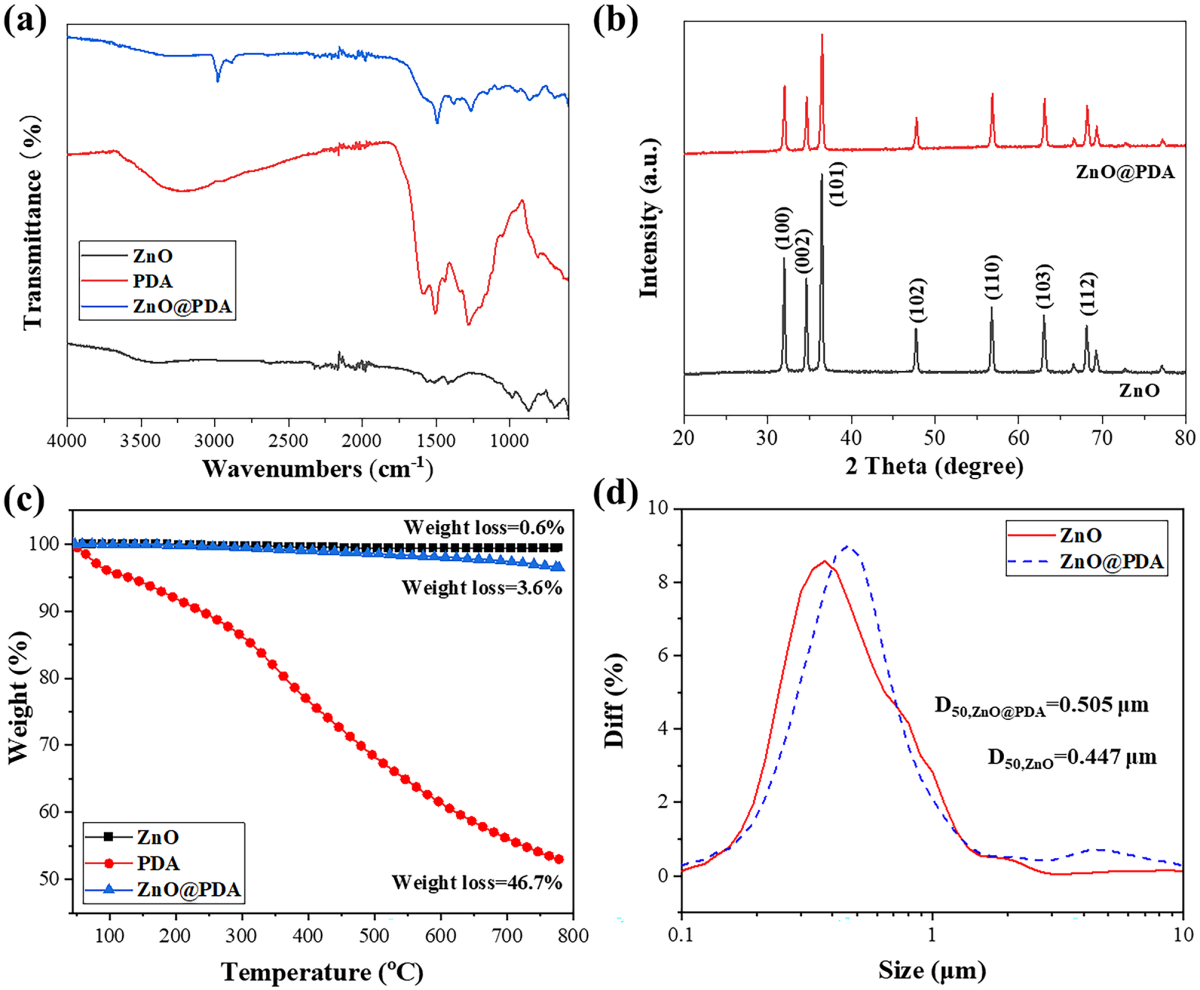
FTIR spectra of ZnO, PDA and ZnO@PDA (**a**), XRD spectra of ZnO and ZnO@PDA (**b**), TGA of ZnO, PDA and ZnO@PDA (**c**), and Particle size distribution of ZnO and ZnO@PDA (**d**)

In order to improve the processing window of the ZnO/PLA composites, the ZnO was first modified with DA to form a PDA coating on the surface. The coating process is shown in Fig. 1 and the detailed procedures were explained in Section “Fabrication of ZnO@PDA particles”. Intuitively, ZnO is a white powder, and the ZnO after PDA coating is gray, as also shown in the right bottom side of Fig. 1. FTIR spectra of ZnO, PDA and ZnO@PDA are shown in Fig. 2a. The polydopamine powder characteristic peaks were observed at 1292 cm^− 1^, 1512 cm^− 1^, 1627 cm^− 1^, and 3379 cm^− 1^ that corresponded to C-O, C = C, C = O and -OH or/and N-H vibrational modes, respectively [25, 26], which are correlated to peaks observed for ZnO@PDA particles, indicating the PDA exists together with ZnO particles.

All the reflection peaks appearing in the XRD have been indexed in Fig. 2b by the reflection planes of (100), (002), (101), (102), (110), (103) and (112). All these peaks of ZnO@PDA remain the same XRD patterns, which are consistent with the ZnO except the slightly reduce of the peak intensity, implying that the surface of ZnO is partially covered by PDA but the structure of ZnO is well-maintained after coating process.

TGA curves of ZnO, PDA and ZnO@PDA are presented in Fig. 2c to investigate the coating content of PDA on ZnO@PDA. It can be seen that the weight loss of the pure ZnO is only 0.6 wt% (800 ^o^C) during the entire heating process, which is mainly derived from the decomposition of the surface organic content or absorbed moisture. By contrast, the weight loss of ZnO@PDA is 3.6 wt% (800 ^o^C), which is derived from the decomposition of PDA. For the pure PDA, the weight loss is 46.7 wt% (800 ^o^C). The coating amount of PDA on the ZnO surface can be calculated according to the following Eq. (2):

2 $${R}_{ZnO@PDA}=x{R}_{PDA}+(1-x){R}_{ZnO}$$

where $x$ represents the coating content (%) of PDA on ZnO@PDA, ${R}_{PDA}$ and ${R}_{ZnO}$ are the char residue of PDA and ZnO, respectively. The coating amount of PDA on ZnO@PDA is calculated with value of 6.5 wt%.

Figure 2d shows the particle size differential of ZnO and ZnO@PDA, which obeys a Gaussian distribution. The shapes of the distribution curves of ZnO are similar to that of ZnO@PDA. D50 represents the particle size at which the cumulative particle size distribution percentage of a sample reaches 50%. Its physical meaning is that the particles with a particle size larger than it account for 50%, and the particles smaller than it also account for 50%. Therefore, D50 is often used to represent the average particle size of the powder. As depicted in Fig. 2d D50 of ZnO@PDA being 0.505 μm, was larger than that of ZnO (0.447 μm). The increase of particle size after modification is because of the successful surface coating.

**Fig. 3:**
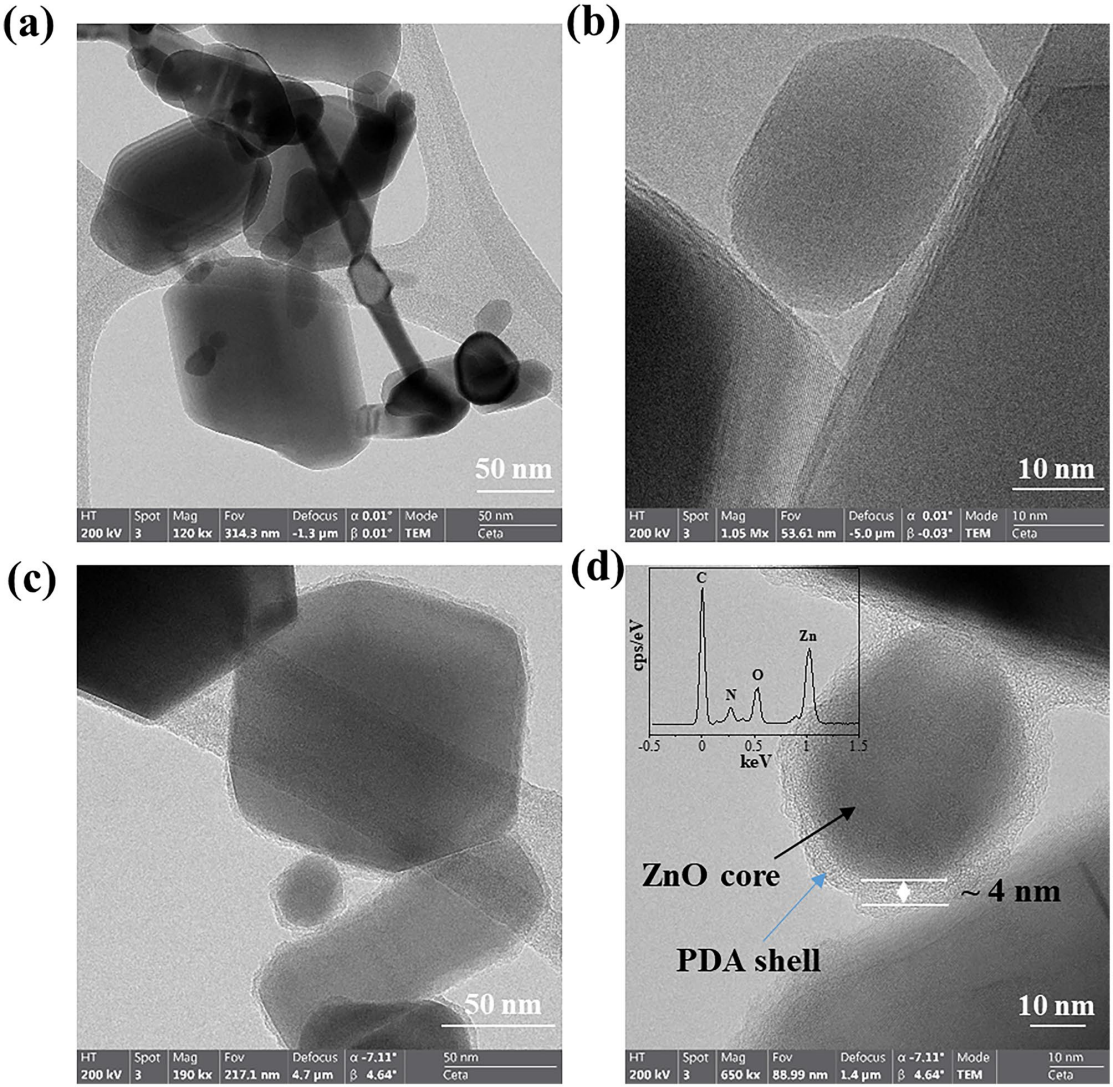
TEM of ZnO (**a**, **b**) and ZnO@PDA (**c**, **d**) (TEM energy spectrum of ZnO@PDA is in the top-left corner of (**d**))

The morphologies of ZnO and ZnO@PDA were observed by TEM. As presented in Fig. 3a, b, ZnO have a hexagonal crystals shape and a clear boundary. For ZnO@PDA, it can be seen from Fig. 3c that ZnO were surrounded by some amorphous substances. Furthermore, the core-shell structure of ZnO@PDA can be clearly observed in the high magnification TEM images of Fig. 3d, which leads to the larger size of ZnO@PDA than pure ZnO (Fig. 2d). It can be known from the measurement that the coating thickness of PDA on the surface of ZnO is about 4 nm. These results directly demonstrated the successful preparation of ZnO@PDA. Figure 3d inset further showed the N signal, which further confirmed the coexistence of PDA.

### Preparation of PLA composites and general properties of the composite materials

**Fig. 4:**
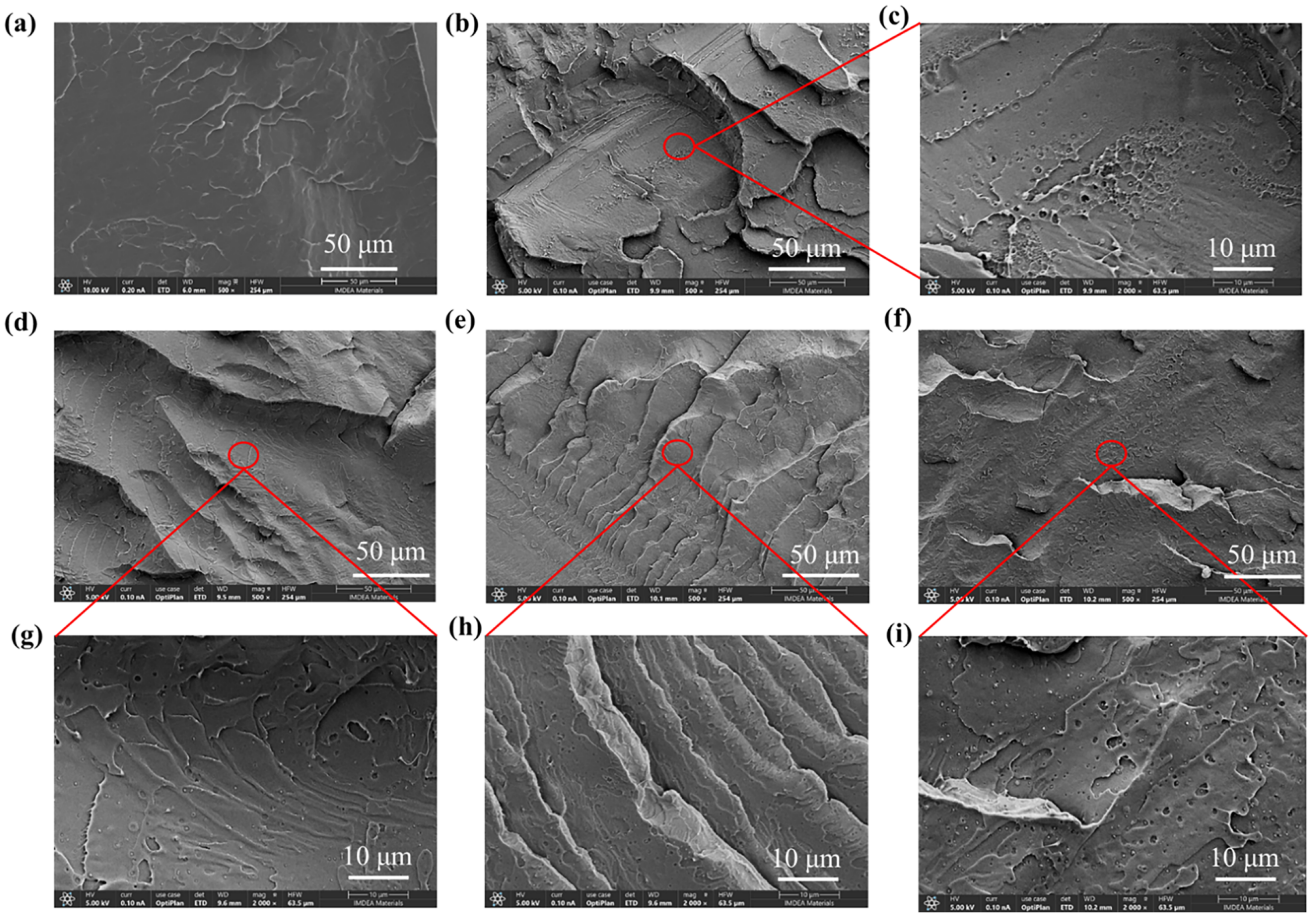
SEM of pure PLA (**a**) and PLA composites (1%-ZnO/PLA (**b**, **c**), 1%-ZnO@PDA/PLA (**d**, **g**), 3%-ZnO/PLA (**e**, **h**) and 3%-ZnO@PDA/PLA (**f**, **i**))

SEM was used to investigate the section morphology of PLA and PLA composites, and the results were shown in Fig. 4. The fracture of pure PLA is relatively flat, showing a brittle fracture (Fig. 4a), while the others were rougher (Fig. 4b, d, e and f). Magnified observation of the dispersion of ZnO in PLA before and after modification (Fig. 4c, g, h and i), no obvious particle aggregation was observed, indicating that ZnO and ZnO@PDA were well dispersed in PLA.

**Fig. 5:**
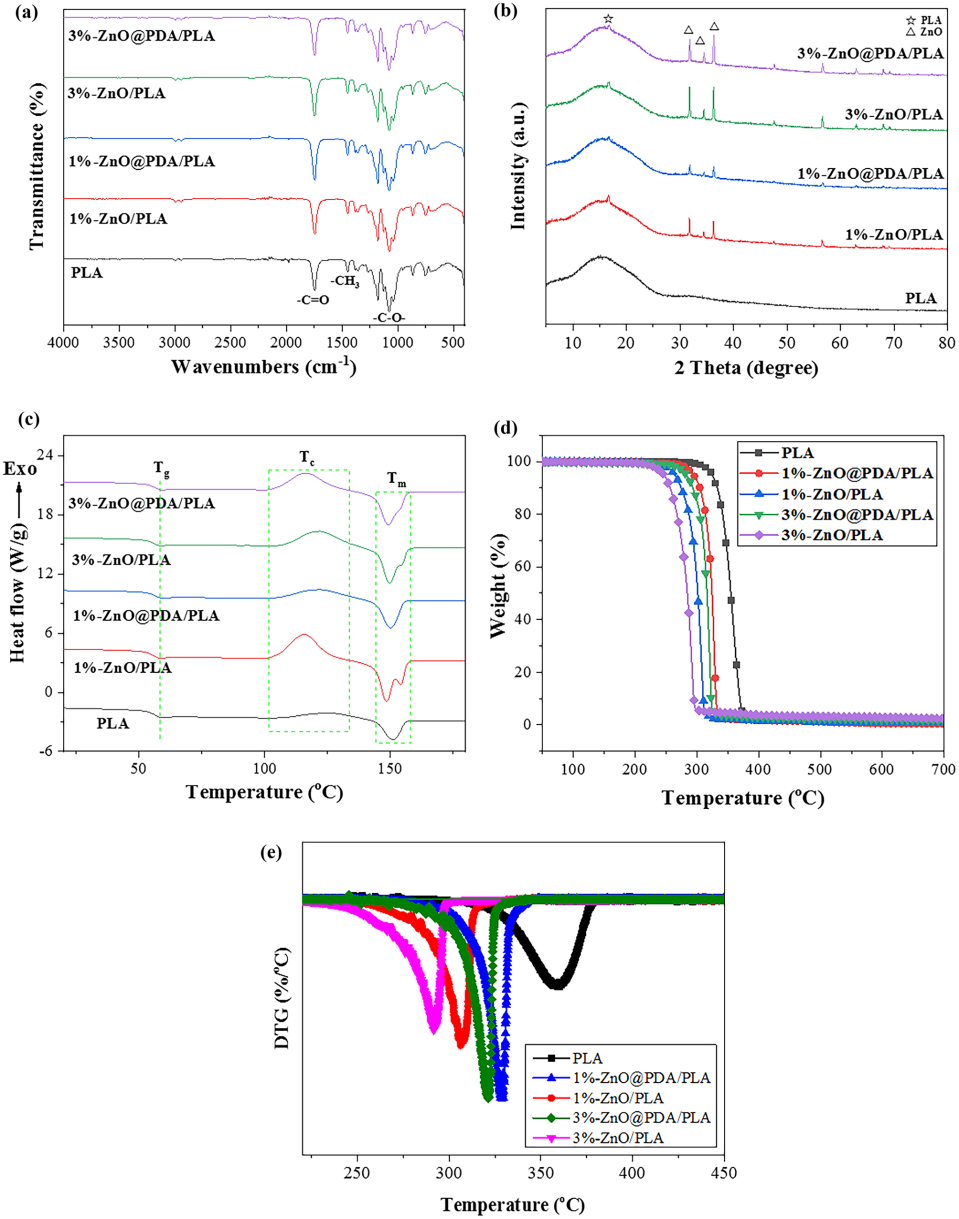
FTIR spectra (**a**), XRD spectra (**b**), DSC curve (**c**), TGA curve (**d**), and DTG curve (**e**) of pure PLA and PLA composites

Figure 5a showed the FTIR spectra of PLA and PLA composites. The C = O stretching of the ester group was attributed as a broad and strong absorption band at 1747 cm^− 1^. The -CH- deformation and asymmetric bands appeared at 1382 cm^− 1^ and 1359 cm^− 1^, respectively. Between 1300 cm^− 1^ and 1000 cm^− 1^, it was possible to observe the asymmetric C-O-C stretching mode at 1179 cm^− 1^, 1128 cm^− 1^, and 1080 cm^− 1^. The curves of PLA composites were similar to the pure PLA.

The effect of ZnO particle content before and after modification on the crystallinity of the PLA were analyzed by XRD. The XRD patterns of PLA and PLA composites are shown in Fig. 5b. Pure PLA showed a broad peaks appearing at approximately 2θ = 10 ~ 20 ^o^, which indicated that the PLA had no polymorphic crystalline transition [27]. However, after adding the fillers, a crystallization peak appeared at 2θ = 16.6 ^o^, which to some extent indicated that the presence of fillers had a positive effect on the crystallization of PLA molecular chain motion. It was further observed from ZnO/PLA composites that with the increasing content of both ZnO and ZnO@PDA (1 and 3 wt%), the intensity of diffraction peaks at approximately 2θ = 31.8 ^o^, 2θ = 34.4 ^o^ and 2θ = 36.3 ^o^ increased, and these diffraction signals were consistent with the standard properties for hexagonal ZnO crystals [28].

The glass transition temperature (T_g_), cold crystallization temperature (T_c_) and melting temperature (T_m_) of both ZnO/PLA and ZnO@PDA/PLA samples were studied by using DSC analysis. The DSC curves and thermal parameters of above samples were presented in Fig. 5c and Table 1, respectively. In the heating and cooling process, pure PLA had no obvious T_c_ peak. But for both ZnO/PLA and ZnO@PDA/PLA samples, the T_c_ peak appeared obviously, which could be because that ZnO or ZnO@PDA can act as a nucleating agent during proper temperature region increasing the molecular chain movability. In addition, compared with pure PLA, the introduction of different contents of ZnO or ZnO@PDA didn’t significantly affect the T_g_ and T_m_ of PLA composites. In terms of the crystallinity, as listed in Table 1, the calculated crystallinities of the composites all slightly increased. This might be due to heterogeneous nucleation of PLA or fillers in PLA acting as a nucleating agent to enhance crystallization of PLA chains [1] during the whole thermal treatment procedure, which is in good agreement with the XRD results in Fig. 5b.

TGA is an important analytical method to measure the changes in the mass of PLA and PLA composites in a variety of processes such as decomposition, degradation, sublimation, vaporization, desorption, oxidation, and reduction [29]. The TGA curves and DTG curves of samples were shown in Fig. 5d, e, respectively. The corresponding parameters were summarized in Table 1. Compared with pure PLA, all modified composites decomposed earlier. But it is worth emphasizing that the thermal decomposition temperature of the ZnO@PDA/PLA samples are all delayed compared with the corresponding ZnO/PLA samples with the same filler content. For example, the thermal decomposition temperature of 1%-ZnO@PDA/PLA at T_5%_, T_50%_, and T_90%_ are 294.2 ^o^C, 324.4 ^o^C and 330.9 ^o^C respectively, generally occurred with relatively higher than 1%-ZnO/PLA (263.9 ^o^C, 301.4 ^o^C and 310.3 ^o^C), indicating that the ZnO@PDA/PLA samples were more stable than that of ZnO/PLA samples. These results showed that the surface modification of ZnO by PDA can effectively alleviate the thermal degradation process of PLA, and can expand the process window of PLA/ZnO composites accordingly.

**Table 1:** Thermal parameters of pure PLA and PLA composites

**Sample**	**T**_**5%**_ **(**^**o**^**C)**	**T**_**50%**_ **(**^**o**^**C)**	**T**_**max**_ **(**^**o**^**C)**	**T**_**90%**_ **(**^**o**^**C)**	**T**_**g**_ **(**^**o**^**C)**	**T**_**c**_ **(**^**o**^**C)**	**T**_**m**_ **(**^**o**^**C)**	$\varDelta {\mathbf{H}}_{\mathbf{c}}$ **(J/g)**	$\varDelta {\mathbf{H}}_{\mathbf{m}}$ **(J/g)**	**φ (%)**
Pure PLA	324.4	354.8	359.3	369.4	56.3	124.9	151.2	10.5	13.9	3.7
1%-ZnO/PLA	263.9	301.4	321.1	310.3	56.3	116.1	148.6	32.6	36.7	4.4
1%-ZnO@PDA/PLA	294.2	324.4	328.2	330.9	56.8	121.9	150.0	20.2	24.2	4.3
3%-ZnO/PLA	246.0	284.4	291.6	295.0	56.2	121.8	149.7	28.9	33.3	4.8
3%-ZnO@PDA/PLA	282.4	316.3	306.7	322.8	56.5	116.9	149.3	28.6	32.4	4.2

### Effective mitigation of PDA coating for processing pyrolysis of PLA composites and 3D printed models

**Fig. 6:**
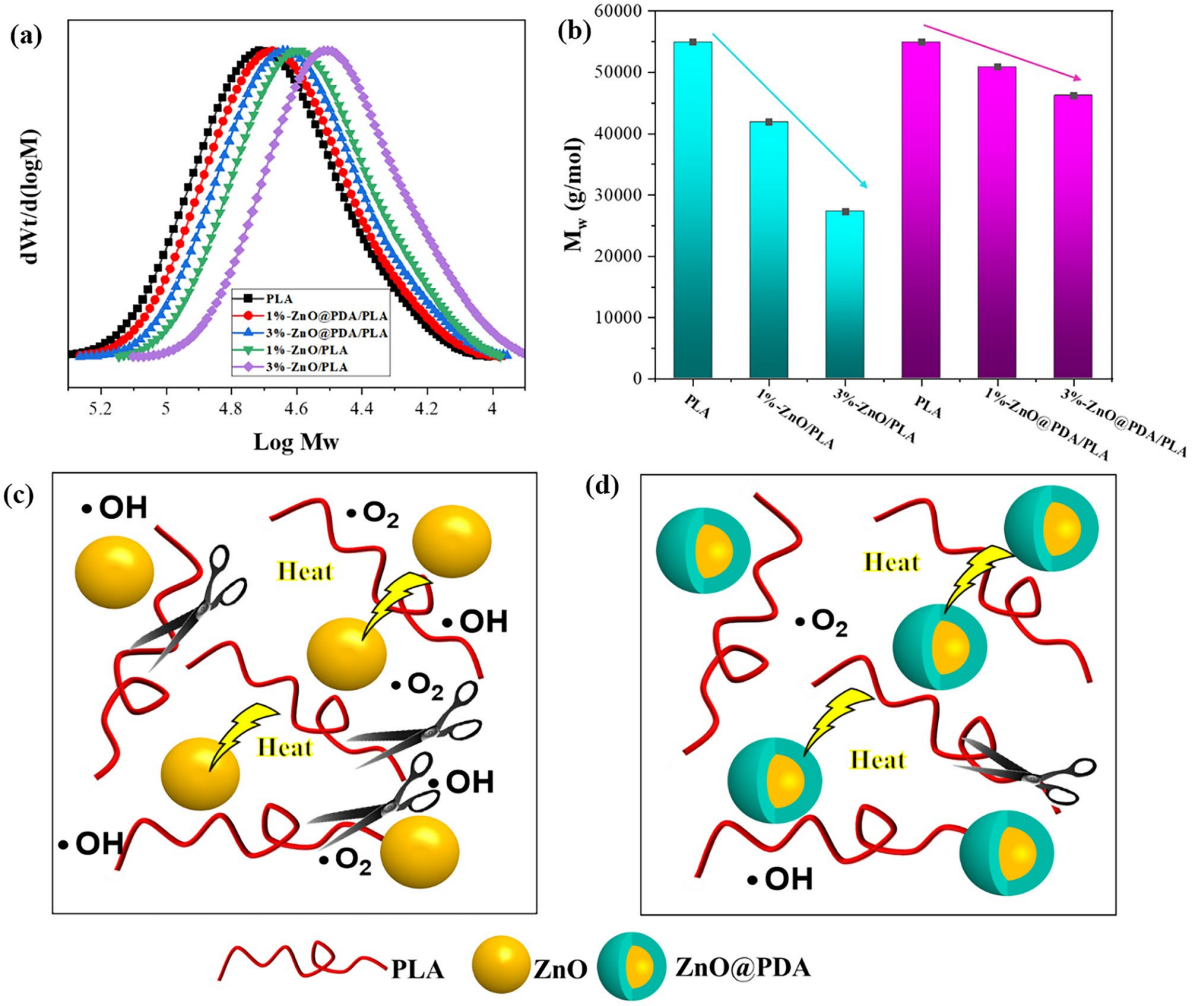
GPC curves (**a**) and bar chart for M_w_ (**b**) of pure PLA and PLA composites, schematic diagram of possible degradation mechanism of ZnO/PLA composites (**c**) and ZnO@PDA/PLA composites (**d**)

The molecular weight of pure PLA and samples with different contents of ZnO and ZnO@PDA after extrusion was measured by GPC, as shown in Fig. 6a and the corresponding data were summarized in Table 2. It can be seen that compared with pure PLA, the M_n_ and M_w_ of ZnO/PLA decrease sharply with the increase of ZnO content, indicating that the addition of ZnO significantly decomposed PLA chains during hot processing. This may be because the exposed ZnO under the action of thermal or oxygen induces the release of oxygen radicals, leading to the degradation and fracture of the PLA chain [30, 31]. However, the degradation degree of ZnO@PDA/PLA is significantly reduced compared to that of ZnO/PLA. M_w_ values in Fig. 6b clearly showed the difference of decreasing rate containing between ZnO and ZnO@PDA containing PLA composites. The degree of molecular weight reduction after adding ZnO@PDA to PLA was much slower than those after adding ZnO, indicating the surface functionalization by PDA could restrain destruction of PLA to some extent.

The schematic diagram of possible degradation mechanism of ZnO and ZnO@PDA were shown in the Fig. 6c, d. During melt processing, the damage of visible light or heat to polymer materials is unavoidable. In fact, the addition of ZnO generates free radicals ⋅O_2_ and ⋅OH and the direct contact between PLA and ZnO leads to accelerated degradation of PLA under visible light and heat conditions severely [32]. While the PDA coating of ZnO@PDA prevented the direct contact between PLA and ZnO (Fig. 6d), which would establish an effective protective pathway for PLA to mitigate relative minor damage.

**Fig. 7:**
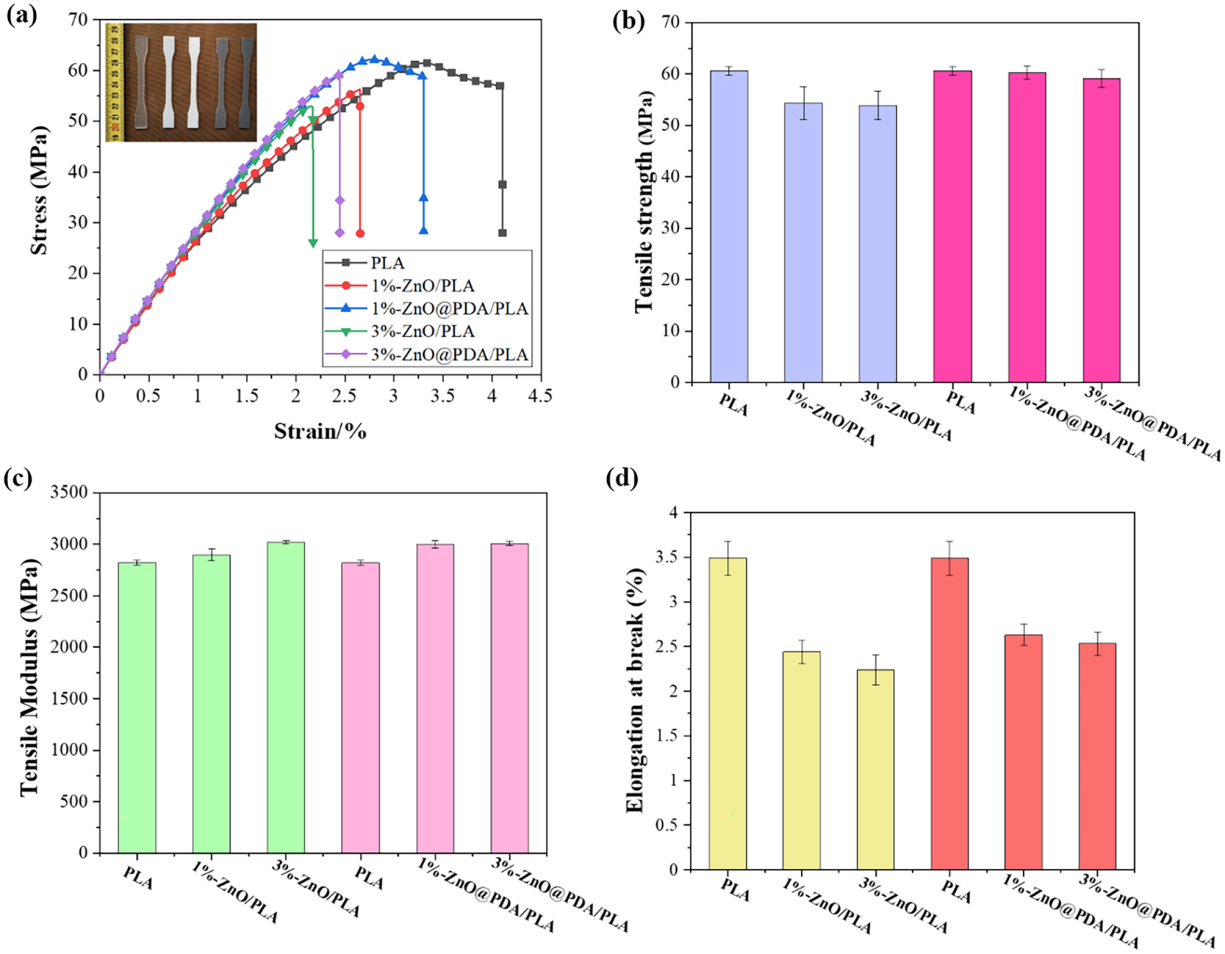
Stress-strain curves (**a**), tensile strength histogram (**b**), tensile modulus histogram (**c**) and elongation at break histogram (**d**) of pure PLA and PLA composites

Mechanical properties of polymer composites were shown in Fig. 7a-d and Table 2. All the modified PLA composites showed enhanced modulus compared with that of pure PLA, which is common phenomenon in the inorganic filler functionalization composites, as widely reported in the literatures elsewhere [1]. From the Fig. 7b and d, we can see that the addition of ZnO reduces the tensile strength and the elongation at break of PLA severely, while ZnO@PDA has relative slight effect on the reduction of tensile strength and elongation at break of PLA. We believe that the decrease in tensile strength and elongation at break of ZnO/PLA or ZnO@PDA/PLA composites is mainly due to the significant decrease in molecular weight of PLA during thermal processing caused by fillers. Among them, ZnO@PDA/PLA composites have better mechanical properties than ZnO/PLA because ZnO@PDA/PLA composites have higher molecular weight than ZnO/PLA with the same filler ratio.

**Fig. 8:**
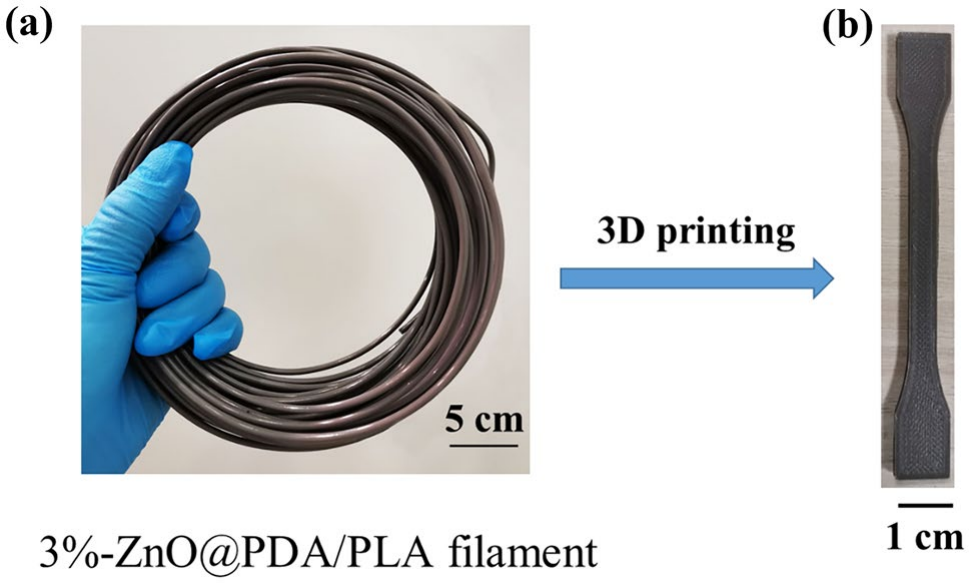
Filaments after 3 devo (**a**) and 3D printed sample (**b**) of 3%-ZnO@PDA/PLA

After precision extrusion in 3devo filament maker, we obtained the filaments of the above five samples. The filaments of the five samples were then expected to be printed by the 3D printer. However, it’s found that the ZnO/PLA filaments occurred multiple fractures when entering the printing channel due to its significantly decreased mechanical strength and flexibility (low elongation %). Fortunately, ZnO@PDA/PLA composites can be printed smoothly for both samples with 1%- and 3%-ZnO@PDA. Figure 8a, b are the filament and 3D printed dog-bone shaped samples of 3%-ZnO@PDA/PLA, as a typical example to proof the 3D printing possibility.

**Table 2:** GPC parameters and mechanical data of the pure PLA and PLA composites

**Sample**	**M**_**w**_ **(g/mol)**	**M**_**n**_ **(g/mol)**	**PDI**	**Tensile strength (MPa)**	**Elongation at break (%)**	**Tensile modulus (MPa)**
Pure PLA	54,885	43,570	1.26	60.57 ± 0.84	3.49 ± 0.19	2821 ± 27
1%-ZnO/PLA	41,861	33,743	1.24	54.30 ± 3.18	2.44 ± 0.13	2897 ± 57
1%-ZnO@PDA/PLA	50,820	40,731	1.25	60.26 ± 1.31	2.63 ± 0.12	3000 ± 39
3%-ZnO/PLA	33,639	27,256	1.23	53.88 ± 2.77	2.24 ± 0.17	3020 ± 14
3%-ZnO@PDA/PLA	46,211	36,884	1.25	59.07 ± 1.71	2.53 ± 0.13	3008 ± 22

### Antimicrobial performance of PLA and PLA composites

**Fig. 9:**
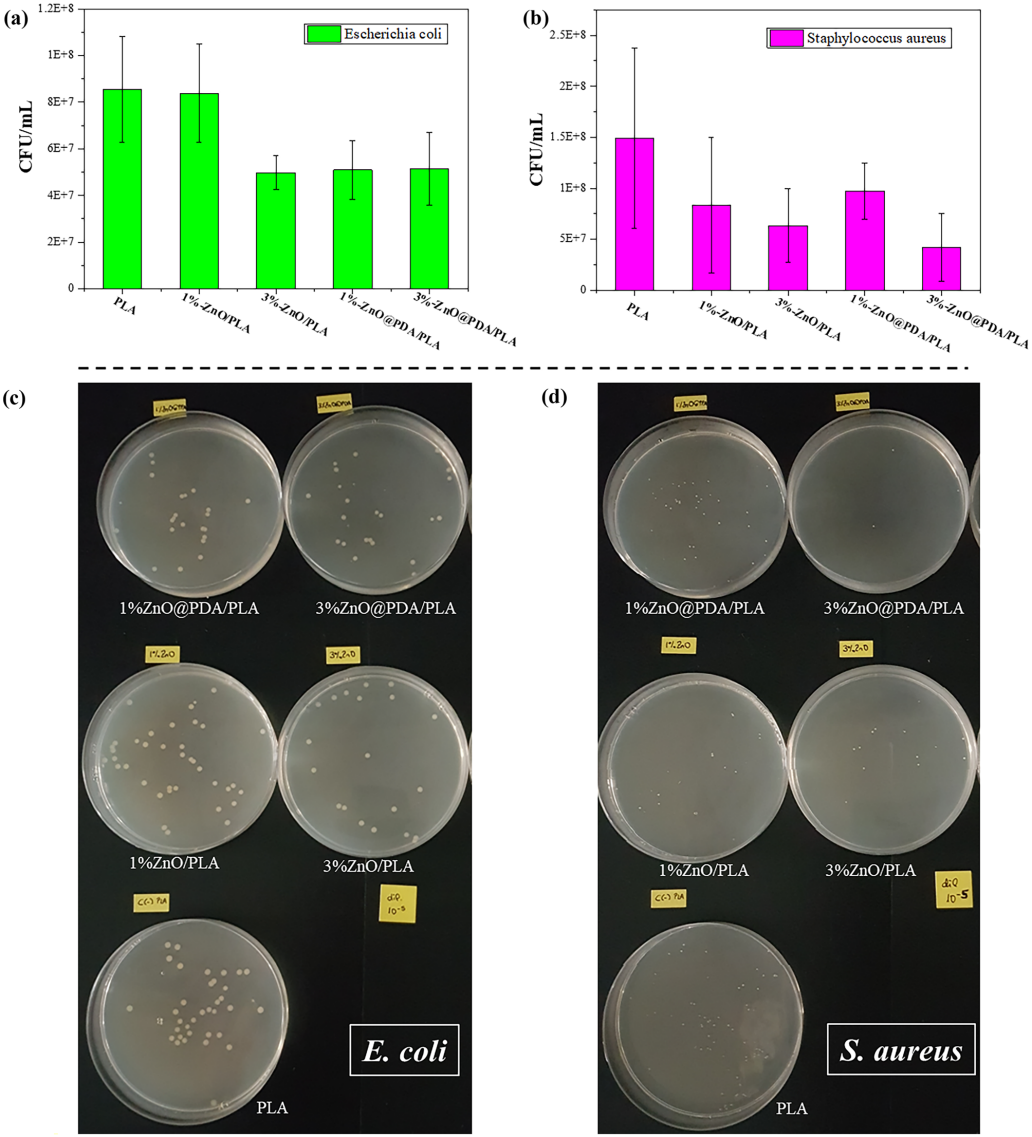
Antimicrobial activity quantification of PLA and PLA composites against Gram-negative bacteria (*E. coli*) (**a**), Gram-positive bacteria (*S. aureus*) (**b**), and formation of bacteria colonies: antimicrobial activity of pure PLA and PLA composites against *E. coli* (**c**) and *S. aureus* (**d**) diluted 10^5^-fold after 24 h of incubation time and recovery of the culture from the material surface

The antimicrobial properties of pure PLA and PLA composites were evaluated according to the ISO 22,196: 2011 as described in Materials and Methods section. The antimicrobial activity of PLA and PLA composites against Gram-negative bacteria (*E. coli*) and Gram-positive bacteria (*S. aureus*) was quantified (Fig. 9a, b) and the formation of bacteria colonies were presented qualitatively in Fig. 9c, d. In the case of *E. coli* (Fig. 9a, c), ZnO/PLA composites exhibit a slight difference in growth after 24 h of incubation compared with that of pure PLA. In general terms (in both ZnO/PLA and ZnO@PDA/PLA composites), there is a decreasing trend in total bacterial growth (p < 0.05 when each composite is compared with pure PLA) and, in the case of 3% ZnO/PLA, the difference is statistically highly significant (p < 0.01). But analyzing the case of *S. aureus* (Fig. 9b and d), statistical analysis results that only 3% ZnO@PDA/PLA has a difference with pure PLA (p < 0.05). This may be because the surface PDA functionalization can improve the contact/interfacial interaction between ZnO@PDA/PLA and the *S. aureus* culture to a certain extent. This differential behavior could be explained due to structural differences between Gram(–) and Gram(+) cell walls.

## Conclusions

In summary, core-shelled ZnO@PDA were constructed by surface functionalization of ZnO with PDA. It was found that the incorporation of ZnO@PDA in PLA at different contents exhibits a dramatic control over the degradation rate when compared to that of the ZnO/PLA with the same filler content. That is to say the introduction of ZnO@PDA can increase the processing window of PLA. Accordingly, ZnO@PDA/PLA samples showed higher tensile strength and elongation at break than that of corresponding ZnO/PLA samples, guaranteeing the possibility of 3D printing. What’s more, the ZnO@PDA/PLA have more bacterial growth disability effect against Gram(+) bacteria than that of pristine PLA. In view of the universal adhesion of PDA and the convenience of synthesis, this method can be a universal particle modification method and is expected to be extended to the surface modification of other functional particles for PLA.

## Data Availability

Not Applicable.
